# Contemporary Management Strategies for Chronic Type B Aortic Dissections: A Systematic Review

**DOI:** 10.1371/journal.pone.0154930

**Published:** 2016-05-04

**Authors:** Arnoud V. Kamman, Hector W. L. de Beaufort, Guido H. W. van Bogerijen, Foeke J. H. Nauta, Robin H. Heijmen, Frans L. Moll, Joost A. van Herwaarden, Santi Trimarchi

**Affiliations:** 1 Thoracic Aortic Research Center, Policlinico San Donato IRCCS, University of Milan, San Donato Milanese, Italy; 2 Department of Vascular Surgery, University Medical Center Utrecht, Utrecht, The Netherlands; 3 Departments of Surgery and Biomedical Engineering, University of Michigan, Ann Arbor, Michigan, United States of America; 4 Department of Cardiothoracic Surgery, St. Antonius Hospital Nieuwegein, Nieuwegein, The Netherlands; University Francisco de Vitoria School of Medicine, SPAIN

## Abstract

**Background:**

Currently, the optimal management strategy for chronic type B aortic dissections (CBAD) is unknown. Therefore, we systematically reviewed the literature to compare results of open surgical repair (OSR), standard thoracic endovascular aortic repair (TEVAR) or branched and fenestrated TEVAR (BEVAR/FEVAR) for CBAD.

**Methods:**

EMBASE and MEDLINE databases were searched for eligible studies between January 2000 and October 2015. Studies describing outcomes of OSR, TEVAR, B/FEVAR, or all, for CBAD patients initially treated with medical therapy, were included. Primary endpoints were early mortality, and one-year and five-year survival. Secondary endpoints included occurrence of complications. Furthermore, a Time until Treatment Equipoise (TUTE) graph was constructed.

**Results:**

Thirty-five articles were selected for systematic review. A total of 1081 OSR patients, 1397 TEVAR patients and 61 B/FEVAR patients were identified. Early mortality ranged from 5.6% to 21.0% for OSR, 0.0% to 13.7% for TEVAR, and 0.0% to 9.7% for B/FEVAR. For OSR, one-year and five-year survival ranged 72.0%-92.0% and 53.0%-86.7%, respectively. For TEVAR, one-year survival was 82.9%-100.0% and five-year survival 70.0%-88.9%. For B/FEVAR only one-year survival was available, ranging between 76.4% and 100.0%. Most common postoperative complications included stroke (OSR 0.0%-13.3%, TEVAR 0.0%-11.8%), spinal cord ischemia (OSR 0.0%-16.4%, TEVAR 0.0%-12.5%, B/FEVAR 0.0%-12.9%) and acute renal failure (OSR 0.0%-33.3%, TEVAR 0.0%-34.4%, B/FEVAR 0.0%-3.2%). Most common long-term complications after OSR included aneurysm formation (5.8%-20.0%) and new type A dissection (1.7–2.2%). Early complications after TEVAR included retrograde dissection (0.0%-7.1%), malperfusion (1.3%–9.4%), cardiac complications (0.0%–5.9%) and rupture (0.5%–5.0%). Most common long-term complications after TEVAR were rupture (0.5%–7.1%), endoleaks (0.0%–15.8%) and cardiac complications (5.9%-7.1%). No short-term aortic rupture or malperfusion was observed after B/FEVAR. Long-term complications included malperfusion (6.5%) and endoleaks (0.0%-66.7%). Reintervention rates after OSR, TEVAR and B/FEVAR were 5.8%-29.0%, 4.3%-47.4% and 0.0%-53.3%, respectively. TUTE for OSR was 2.7 years, for TEVAR 9.9 months and for B/FEVAR 10.3 months.

**Conclusion:**

We found a limited early survival benefit of standard TEVAR over OSR for CBAD. Complication rates after TEVAR are higher, but complications after OSR are usually more serious. Initial experiences with B/FEVAR show its feasibility, but long-term results are needed to compare it to OSR and standard TEVAR. We conclude that optimal treatment of CBAD remains debatable and merits a patient specific decision. TUTE seems a feasible and useful tool to better understand management outcomes of CBAD.

## Introduction

Currently, the optimal management of chronic type B aortic dissections (CBAD) remains undetermined as there have been no randomized controlled trials comparing open surgical repair (OSR) and thoracic endovascular aortic repair (TEVAR) [1]. Furthermore, branched and fenestrated TEVAR (B/FEVAR) are emerging as new techniques to treat more complicated cases with an endovascular approach [2]. The initial treatment objective for uncomplicated acute type B dissections is clinical stabilization of the patient through optimal medical therapy (OMT) to prevent propagation of the dissection, malperfusion, rapid aortic dilatation and/or rupture. However, secondary interventions after initial OMT are common, with intervention rates ranging between 9.0% and 40.6% [3–14]. Most common indications for secondary interventions for CBAD include aneurysm formation, rapid aneurysmal sac enlargement, extension of dissection and malperfusion [3–13, 15].

Both endovascular therapies and OSR show up- and downsides; endovascular management is less invasive, however successful treatment during the chronic phase may be challenging due to thickening of the intimal flap. Standard TEVAR for CBAD patients has shown acceptable mid-term outcomes, however complete aortic remodeling was seen in only 36% of cases [16], mostly precluded due to abdominal extension of the dissection. Such extended involvement determines a thoracoabdominal aortic issue that may require a more extensive repair. In such a setting, branched and fenestrated procedures may offer an endovascular solution. However, anatomical limitations like narrow lumens and technical difficulties, such as the orientation of the branches and fenestrations, and the presence of the lamella inside the lumen, make the procedure challenging. Nevertheless, in general, any type of endovascular management could be of value in chronic patients, reducing operative risks of OSR. An open approach is more invasive with higher operative risks [17], but unlike endovascular management, it is rarely affected by anatomical constraints. Currently, OSR is the most commonly adopted strategy, in particular in younger patients and those affected by connective tissue disorders, while endovascular treatment has been adopted for specific clinical scenarios such as limited extent of the dissection, intramural hematoma evolving with penetrating aortic ulcer, and older patients. Our aim was to systematically review the literature and compare outcomes of CBAD patients managed with OSR, TEVAR and B/FEVAR, who were initially treated with OMT alone.

## Materials and Methods

### Search strategy

The EMBASE and MEDLINE databases were searched for eligible studies from January 1^st^, 2000 up to October 1^st^, 2015. The following search terms were used: ‘follow-up’, ‘chronic’, ‘post-dissection’ ‘type B’, ‘aortic/aorta dissection’ and ‘outcome’, or synonyms (S1 Appendix).

### Article selection

The Meta-analysis of Observational Studies in Epidemiology (MOOSE) guidelines were used for analysis of the studies in this systematic review [18]. Included studies were critically assessed for study design, heterogeneity, possible bias, and other limitations. Two reviewers (AK and HB) performed eligibility for this systematic review independently. Disagreement between reviewers was resolved during a consensus meeting. Inclusion criteria were: (1) English language; (2) case series describing outcomes of OSR, TEVAR, B/FEVAR or multiple, for CBAD; and (3) follow-up of at least one year. Exclusion criteria were: (1) studies before 2000 to ensure contemporary practice in all included studies; (2) patients initially treated with invasive therapy; (3) case reports; (4) studies describing mixed populations without separate outcomes listed; and (5) articles from the same institution or author were studied critically to ensure no overlapping patient populations were included in the final analysis.

### Extracted data and endpoints

Data extracted included demographics, patient history, intervention details, and follow-up outcomes. The primary endpoints were early mortality, and one- and five-year survival. Secondary endpoints included the occurrence of complications. Early outcomes were defined as either in-hospital or 30-day outcome. Long-term outcomes were defined as occurring during follow-up. Rapid aortic enlargement was defined as ≥0.5 cm increase in diameter per year.

### TUTE

The concept of “time until treatment equipoise” (TUTE) has been described in an attempt to better and easier advise patients of relative risks of different management modalities [19]. It is defined as the duration of time that elapses after an intervention, before the risk of the intervention is canceled out and reversed by the cumulative risk of conservative management. In other words, it is the point in time during follow-up after which an intervention is most beneficial, because the mortality risk of the intervention is lower than the mortality risk of continuing conservative management. TUTE may guide decision making for asymptomatic patients on prognostic grounds. The equipoise is set at the point where the areas between the survival curves of no intervention and intervention are equal. This point is chosen instead of the crossing of the lines, because the intervention itself also carries operative mortality risk, which needs to be accounted for. To estimate TUTE for OSR, TEVAR and B/FEVAR, we performed a TUTE analysis as described by the creators of the concept [19]. In this analysis, the gender, age, mortality rate for the intervention and the expected added mortality rate per year due to the natural history of the condition, are entered. We used the mean age for each of the interventions and the 30-day mortality rate for each intervention. The expected mortality without intervention was adopted from recent available literature [20]. Based on these risk factors, survival curves are calculated, and the point in time where the area between the two curves before and after the crossing of the lines is equal (intervention vs. no intervention) is given. This is the point in time after which an intervention improves survival compared to only medical management, e.g. TUTE.

### Statistical analysis

We discussed the end-points and our rationale for this study with our institutional statistical center (Julius Support Center, UMC Utrecht, the Netherlands). After initial investigation of available studies, it was concluded that a meta-analysis was not feasible and not advisable. This decision was made because of the large heterogeneity among the available literature, since all studies used different in- and exclusion criteria, diverse definitions, and reported different follow-up times. Furthermore, in many studies the original data were not present.

Data are presented as mean ± SD or as percentage. Percentages per variable were calculated by dividing the observed total by the combined total of patients from the studies reporting the characteristic. Values of <0.05 were considered significant. Data analysis and graphing were performed using Microsoft Excel (Microsoft Inc.) and SPSS (SPSS 22 Inc., Chicago, Ill, USA).

## Results

### Included studies

**Fig 1:**
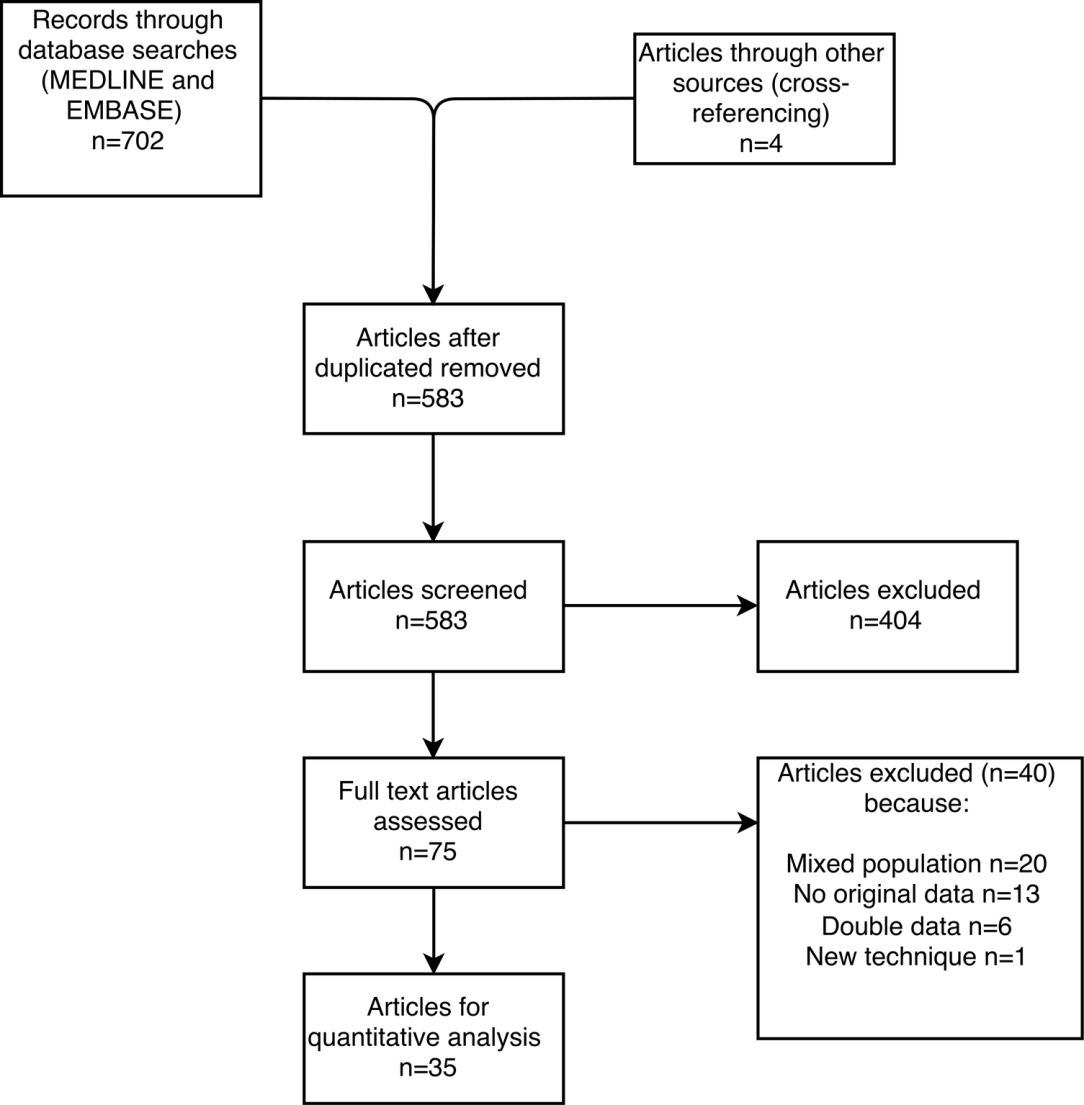
Flowchart of article selection.

A total of 35 articles were selected for systematic review (Fig 1). The initial search of EMBASE and MEDLINE provided 702 articles. After removal of duplicates, 579 articles remained. Of these, 404 articles could be excluded based on the content of the abstract. Seventy-one full-text articles were assessed more closely, after which another 40 articles were excluded. Thirty-one articles were deemed eligible for this systematic review. Cross-referencing of the remaining articles yielded four articles, leaving a final number of 35 articles. No qualitative analysis, e.g. meta-analysis, was performed since the heterogeneity of included studies was large, and therefore a quantitative analysis was most suitable.

### Patient characteristics

The OSR cohort comprised of 1081 patients, with a mean age of 58.2 ± 3.8 years [21–30]. The reported follow-up for these studies ranged between 34 months and 102 months. Overall, there were 816 males (74.2%) [21–31]. Demographic details and medical history of these patients are listed in S1 Table.

The TEVAR cohort consisted of 1397 patients with a mean age of 59.4 ± 4.2 years and 76.0% was male (n = 1051) [21, 29, 32–52]. Follow-up ranged between 12 and 90 months. All TEVAR patient characteristics are shown in S2 Table. Sixty-one B/FEVAR patients were included, mean age 65.7 (± 8.0 years) [53, 54]. Follow-up ranged between 17 months and 20.4 months. Overall, 83.6% of patients were males. All other details are listed in S3 Table.

### Interventional details

Several studies described the timing of OSR; elective interventions were performed between 53.2% and 95.7% [21–25, 30], while urgent and emergent interventions were performed between 10.5% and 12.5% [21, 24], and 3.1% to 7.7% [21, 25, 30], respectively. The exact interval between incident dissection and OSR was available in three studies, ranging between a mean of 32.4–61.0 months [29, 30] and a median of 31.0 (thoraco-abdominal extent) to 43.0 months (limited to descending aorta) [21]. Mean time to intervention was 35.5 months. For 37.4% of the patients the intervention was limited to the descending aorta [21–25, 29, 30], while for the remaining patients the intervention was thoraco-abdominal [21–25, 27, 29, 30]. The operative details are reported in S1 Table.

For TEVAR, elective interventions ranged between 62.5% and 100.0% [21, 28, 38, 45, 46, 52], urgent operations between 18.8% and 97.2% [21, 28, 41], and emergency procedures were performed between 2.0% and 18.8% [21, 28, 41, 43, 45, 46, 49, 51, 52]. Reported time intervals from incident dissection to TEVAR ranged between a median of 3 weeks up to 36.0 months [21, 34, 36, 44, 46] and a mean of 3 weeks and 53.8 months [29, 33, 37, 38, 40, 43, 45, 49, 50]. Mean time to intervention was 24.4 months. Indications for TEVAR were aortic aneurysm (74.5% to 100.0%) [28, 32, 35, 37, 43, 45, 48, 49], failure of OMT (12.3%) [32], rupture (2.7% to 10.0%) [32, 35, 41, 43, 46, 49], rapid aortic enlargement (11.8% to 100.0%) [28, 37, 41, 43, 49], recurrent/refractory pain (4.3% to 57.7%) [32, 37, 41, 43, 49], malperfusion (2.5% to 18.8%) [32, 41, 46, 49], patent false lumen (FL) (64.2%) [40] and other indications (6.6% to 23.5%) [37, 40, 49]. Double indications could be present in a single patient. Complete details are listed in S2 Table.

All B/FEVAR procedures were elective and the only reported indication for these procedures was aneurysmal degeneration [53, 54]. Reported time from incident dissection to the intervention was noted in one study and was 31.0 months [54]. Technical success was achieved in 93.5% in one case series [54] and 100.0% in another study [53]. Complete details are listed in S3 Table.

### Survival

Short-term mortality after OSR ranged between 5.6% and 21.0% [21–30]. One-, five- and ten-year survival was between 72.0% and 92.0% [21, 23, 24, 27, 28, 30], 53.0% and 86.7% [22–25, 27, 29], and between 32.0% and 60.0% [23, 25, 27], respectively. One study reported a 15-year survival of 49.0% [25] (Table 1).

**Table 1:** Complications and survival OSR.

	Andersen 2014[21]	Bashir 2014[22]	van Bogerijen 2015[29]	Conrad 2011[23]	Conway 2014[24]	Estrera 2015[25]	Fujikawa 2015 [30]	Goksel 2008[26]	Kouchoukos 2015[27]	Nozdrzykowski 2013[28]	Safi 2002[31]
ST Stroke	5 (15.6)	7 (11.3)	1 (1.1)	2 (2.7)	2 (2.3)	5 (2.4)	7 (3.0)	0	2 (2.9)	2 (13.3)	NR
ST SCI	3 (9.4)	2 (3.2)	4 (4.4)	12 (16.4)	2 (2.3)	NR	14 (6.0)	0	4 (5.8)	2 (13.3)	7 (3.6)
ST ARF	3 (9.4)	16 (26.0)	7 (7.8)	8 (11.0)	2 (2.3)	21 (10.0)	24 (10.2)	0	3 (4.3)	5 (33.3)	NR
ST MI	NR	NR	NR	10 (14.0)	NR	NR	9 (3.8)	0	2 (2.9)	NR	NR
FU Rupture	NR	NR	NR	NR	NR	NR	1 (0.4)	0	NR	NR	NR
FU Malperfusion	NR	NR	NR	NR	NR	NR	NR	0	NR	NR	NR
FU aneurysm formation	NR	NR	6 (6.7)	NR	5 (5.8)	NR	NR	3 (20.0)	NR	NR	NR
FU new type A dissection	NR	NR	2 (2.2)	NR	NR	NR	4 (1.7)	NR	NR	NR	NR
Reoperation	4 (12.5)	7 (11.3)	10 (11.1)	NR	5 (5.8)	NR	31 (13.0)	3 (20.0)	20 (29.0)	5 (33.3)	NR
ST mortality	2 (6.3)	13 (21.0)	5 (5.6)	8 (11.0)	5 (5.8)	18 (18.6)	20 (8.5)	2 (13.3)	4 (5.8)	2 (13.3)	NR
1-year survival	88.0	NR	NR	72.0	92.0	NR	87.6	NR	86.6	73.3	NR
3-y survival	NR	NR	NR	NR	NR	NR	86.5	NR	NR	73.3	NR
5-year survival	NR	72.6	86.7	53.0	83.0	72.0	NR	NR	65.0	NR	NR
7-year survival	NR	NR	NR	NR	70.0	NR	NR	NR	NR	NR	NR
10-year survival	NR	NR	NR	32.0	NR	60.0	NR	NR	40.0	NR	NR

ARF = acute renal failure; FU = follow-up; MI = myocardial infarction; OSR = open surgical repair; SCI = spinal cord ischemia; ST = short term

For TEVAR, early mortality was reported to be between 0.0% and 13.7% [21, 28, 29, 32–52]. One-year survival was between 82.9% and 100.0% [21, 28, 32, 35, 37, 40, 41, 43, 47]. Five-year survival was between 64.0% and 88.9% [29, 40, 41, 43, 45, 47, 50, 52]. Ten-year survival was reported in one study, with a survival rate of 63.0% [40] (Table 2). One-year dissection-related survival was 97.1% in one study [41] and five-year dissection-related survival ranged between 92.1% and 98.3% [39, 41, 45, 52].

**Table 2:** Complications and survival TEVAR.

	Andacheh 2012[32]	Andersen 2014[21]	van Bogerijen 2015[29]	Chen 2013[33]	Czerny 2010[34]	Guangqi 2009[35]	Jia 2013[36]	Kang 2011[37]	Kato 2002[38]	Kim 2009[39]	Kitamura 2014[40]	Lee 2013[41]	Melissano 2008[42]	Nathan 2015[43]	Nozdrzykowski 2013[28]	Oberhuber 2011[44]	Parsa 2011[45]	Patterson 2013[52]	Sayer 2008[46]	Scali 2013[47]	Shimono 2002[48]	Song 2006[49]	Xu 2010[50]	Yang 2012[51]
TEVAR	73	44	32	56	14	49	208	76	14	72	53	71	11	47	32	19	51	196	40	80	13	17	84	28
ST Stroke	1 (1.4)	0	1 (3.1)	3 (5.4)	0	1 (2.0)	0	NR	0	0	2 (3.8)	NR	0	2 (4.3)	1 (3.1)	0	0	4 (2.1)	NR	8 (10.0)	NR	2 (11.8)	NR	1 (3.6)
ST SCI	1 (1.4)	0	0	0	0	0	2 (1.0)	NR	NR	NR	0	NR	0	3 (6.4)	3 (9.3)	2 (10.5)	0	6 (3.1)	0	10 (12.5)	NR	NR	NR	NR
ST ARF	NR	1 (2.3)	1 (3.1)	4 (7.1)	0	0	NR	NR	NR	1 (1.4)	1 (1.9)	NR	1 (9.1)	3 (6.4)	11 (34.4)	NR	1 (2.0)	NR	0	1 (1.3)	NR	1 (5.9)	2 (2.4)	2 (7.1)
ST retro diss	4 (5.5)	NR	NR	1 (1.8)	0	NR	2 (1.0)	1 (1.3)	NR	NR	NR	NR	0	0	NR	NR	2 (3.9)	NR	1 (2.5)	NR	NR	1 (5.9)	1 (1.2)	2 (7.1)
ST malperfusion	NR	NR	NR	NR	NR	NR	NR	NR	NR	NR	NR	NR	NR	1 (2.1)	3 (9.4)	NR	NR	NR	NR	1 (1.3)	NR	NR	NR	1 (3.6)
ST cardiac complications	4 (5.5)	NR	NR	NR	1 (7.1)	NR	2 (1.0)	NR	NR	NR	NR	NR	0	NR	NR	NR	NR	NR	NR	3 (3.8)	NR	1 (5.9)	NR	NR
ST rupture	2 (2.7)	NR	NR	NR	NR	NR	NR	NR	NR	NR	NR	NR	NR	1 (2.1)	NR	NR	NR	1 (0.5)	2 (5.0)	NR	NR	NR	NR	NR
FU Rupture	NR	NR	NR	NR	1 (7.1)	1 (2.0)	6 (2.9)	NR	NR	NR	2 (3.8)	1 (1.4)	NR	NR	2 (6.3)	NR	NR	1 (0.5)	NR	NR	NR	NR	NR	NR
FU Malperfusion	NR	NR	NR	2 (3.6)	NR	NR	NR	1 (1.3)	0	NR	NR	NR	NR	NR	NR	NR	NR	7 (3.6)	NR	NR	NR	NR	NR	NR
FU Endoleak	7 (9.6)	2 (4.5)	4 (12.5)	1 (1.8)	2 (14.3)	5 (10.2)	3 (1.4)	5 (6.6)	0	6 (8.3)	NR	1 (1.4)	NR	NR	2 (6.3)	3 (15.8)	2 (3.9)	12 (6.1)	NR	NR	NR	NR	7 (8.3)	4 (14.3)
FU cardiac compl	NR	NR	NR	NR	NR	NR	NR	NR	NR	NR	NR	NR	NR	NR	NR	NR	NR	NR	NR	NR	NR	1 (5.9)	NR	NR
Stent collapse/migration	NR	1 (2.3)	NR	NR	NR	NR	NR	3 (3.9)	NR	NR	NR	2 (2.8)	NR	NR	1 (3.1)	NR	NR	NR	NR	NR	NR	NR	NR	NR
Reoperation	11 (15.1)	14 (31.8)	5 (15.6)	NR	2 (14.3)	NR	9 (4.3)	19 (25.0)	NR	9 (12.5)	NR	25 (35.2)	NR	9 (19.1)	9 (28.1)	9 (47.4)	5 (9.8)	34 (17.4)	6 (15.0)	23 (28.8)	NR	3 (17.6)	3 (3.6)	NR
ST mortality	10 (13.7)	0	0	0	0	4 (8.2)	0	4 (5.3)	0	0	0	1 (1.4)	0	2 (4.3)	2 (6.3)	0	0	8 (4.1)	3	2	0	2	1	2
1-year survival	86.0	90.0	NR	NR	NR	82.9	NR	86.0	NR	NR	100.0	97.1	NR	91.5	87.5	NR	NR	NR	NR	89.0	NR	NR	NR	NR
1-y aorta survival	NR	NR	NR	NR	NR	NR	NR	NR	NR	NR	NR	97.1	NR	NR	NR	NR	NR	NR	NR	NR	NR	NR	NR	NR
5- year survival	NR	NR	78.1	NR	NR	NR	NR	NR	NR	NR	86.0	88.9	NR	89.0	NR	NR	77.0	64.0	NR	70.0	NR	NR	84.4	NR
5-year aorta survival	NR	NR	NR	NR	NR	NR	NR	NR	NR	98.3	NR	92.1	NR	NR	NR	NR	98.0	96.0	NR	NR	NR	NR	NR	NR
10-y survival	NR	NR	NR	NR	NR	NR	NR	NR	NR	NR	63.0	NR	NR	NR	NR	NR	NR	NR	NR	NR	NR	NR	NR	NR

ARF = acute renal failure; FU = follow-up; MI = myocardial infarction; SCI = spinal cord ischemia; ST = short term; TEVAR = thoracic endovascular aortic repair

Early mortality after B/FEVAR ranged between 0.0% and 9.7% [53, 54]. One-year survival was between 76.4% and 100.0% [53, 54]. Five-year survival was not available in these studies. Two- and three-year survival was noted in one study, being 85.0%-100.0% and 75.0%-85.0%, respectively [53]. All details can be found in Table 3.

**Table 3:** Complications and survival B/FEVAR.

	Kitagawa 2013^a^ [53]	Kitagawa 2013^b^ [53]	Oikonomou 2014 [54]
B/FEVAR	15	15	31
ST Stroke	0	0	0
ST SCI	0	0	4 (12.9)
ST ARF	0	0	1 (3.2)
ST retro diss	NR	NR	NR
ST malperfusion	0	0	0
ST cardiac complications	NR	NR	1 (3.2)
ST rupture	0	0	0
FU Rupture	0	0	0
FU Malperfusion	0	0	2 (6.5)
FU Endoleak	10 (66.7)	0	12 (38.7)
FU cardiac compl	NR	NR	NR
Stent collapse/migration	NR	NR	NR
Reoperation	8 (53.3)	0	7 (22.6)
ST mortality	0	0	2 (9.7)
1-year survival	85.0	100.0	76.4
2-year survival	85.0	100.0	NR
3-year survival	85.0	75.0	NR

ARF = acute renal failure; B/FEVAR = branched and fenestrated thoracic endovascular aortic repair; FU = follow-up; MI = myocardial infarction; SCI = spinal cord ischemia; ST = short term

^a^ Extensive dissection cohort (Type II/III)

^b^ Focal dissection cohort (without visceral involvement)

Three studies reported outcomes for both OSR and TEVAR. These studies might be of most predictive value and most informative, since they compared both interventions using a similar population of patients. No differences were reported for one-year survival between OSR and TEVAR [21, 28], as well as for five-year survival (p-value 0.23) [29].

### Complications

For OSR, the most common early complications were stroke (0.0% and 13.3%) [21–30], spinal cord ischemia 0.0%-16.4%) [21–24, 26–31] and acute renal failure (0.0% -33.3%). Long-term complications after OSR included aneurysm formation (5.8%-20.0%) [24, 26, 29] and new type A dissection (1.7–2.2%) [29, 30]. Complete results are shown in Table 1.

Most common early complications after TEVAR included stroke (0.0%–11.8%) [21, 28, 29, 32–36, 38–40, 42–45, 47, 49, 51, 52], spinal cord ischemia (0.0%–12.5%) [21, 28, 29, 32–36, 40, 42–47, 52] and acute renal failure (0.0%–34.4%) [3, 21, 28, 29, 33–35, 39, 40, 42, 43, 45–47, 49–51]. Endoleaks (0.0%–15.8%) [21, 28, 29, 32–39, 41, 44, 45, 50–52] were common during follow-up. Other late or long-term complications included rupture (0.5%–7.1%) [28, 34–36, 40, 41, 52], malperfusion (0.0%–3.6%) [33, 37, 38, 52] and cardiac complications (5.9%-7.1%) [34, 49]. All results are listed in Table 2.

Early complications after B/FEVAR included spinal cord ischemia (0.0%-12.9%) [21, 28, 29, 32–36, 40, 42–47, 52–54], acute renal failure (0.0%–3.2%) [53, 54], and cardiac complications (3.2%) [54]. Late complications included malperfusion (0.0%–6.5%) and endoleaks (0.0%–66.7%) [53, 54]. All results are listed in Table 3.

### Reinterventions

Reoperations after OSR were common, ranging between 5.8% and 29.0% (Table 1) [21, 22, 24, 26–30]. Most reinterventions after OSR were managed with another open repair; only in a small number of cases an endovascular procedure was performed [21, 22, 24, 26–30]. Reasons for reintervention included retrograde dissection [29], bleeding [22], aneurysm formation [24, 29] and renal failure [29]. One study described mortality after the reintervention (30.0%). Indications for these procedures were graft infection, thoraco-abdominal aneurysm and aneurysmal growth of distal dissection. Procedures performed were TEVAR (n = 2) and hybrid (n = 1) [29].

For TEVAR, reinterventions were reported between 4.3% and 47.4% (Table 2) [21, 28, 29, 32, 34, 36, 37, 39, 41, 43–47, 49, 50, 52]. Reported reinterventions were TEVAR (58.9%), OSR (27.0%), embolization/ballooning (8.0%) or other (6.1%). Common reasons for reintervention included endoleak [21, 29, 32, 36, 37, 39, 44–46, 50, 52], aneurysm formation [21, 34, 37, 46, 49, 52], retrograde dissection [21, 29, 37, 46, 49, 52], distal FL perfusion [21, 32, 44, 49, 52], rupture [34, 36, 52], and malperfusion syndromes [52]. Only a few studies described outcomes after secondary intervention: Andacheh et al. reported no mortality [32], while van Bogerijen reported two deaths due to type III endoleaks (40.0%) [29]. Jia et al. reported a mortality of 66.6% after secondary intervention; reasons for mortality were multi-organ failure (n = 1), type A dissection (n = 1) and unknown (n = 4) [36]. Lastly, Nathan et al. reported one death after open surgical reintervention (11.1%) [43].

Reinterventions after B/FEVAR were between 0.0% and 53.3% (Table 3) [53, 54]. Reported reinterventions were all endovascular, always for treating endoleaks. Only one study described outcomes after secondary intervention, with no observed mortality [54].

### Time until Treatment Equipoise (TUTE)

**Fig 2:**
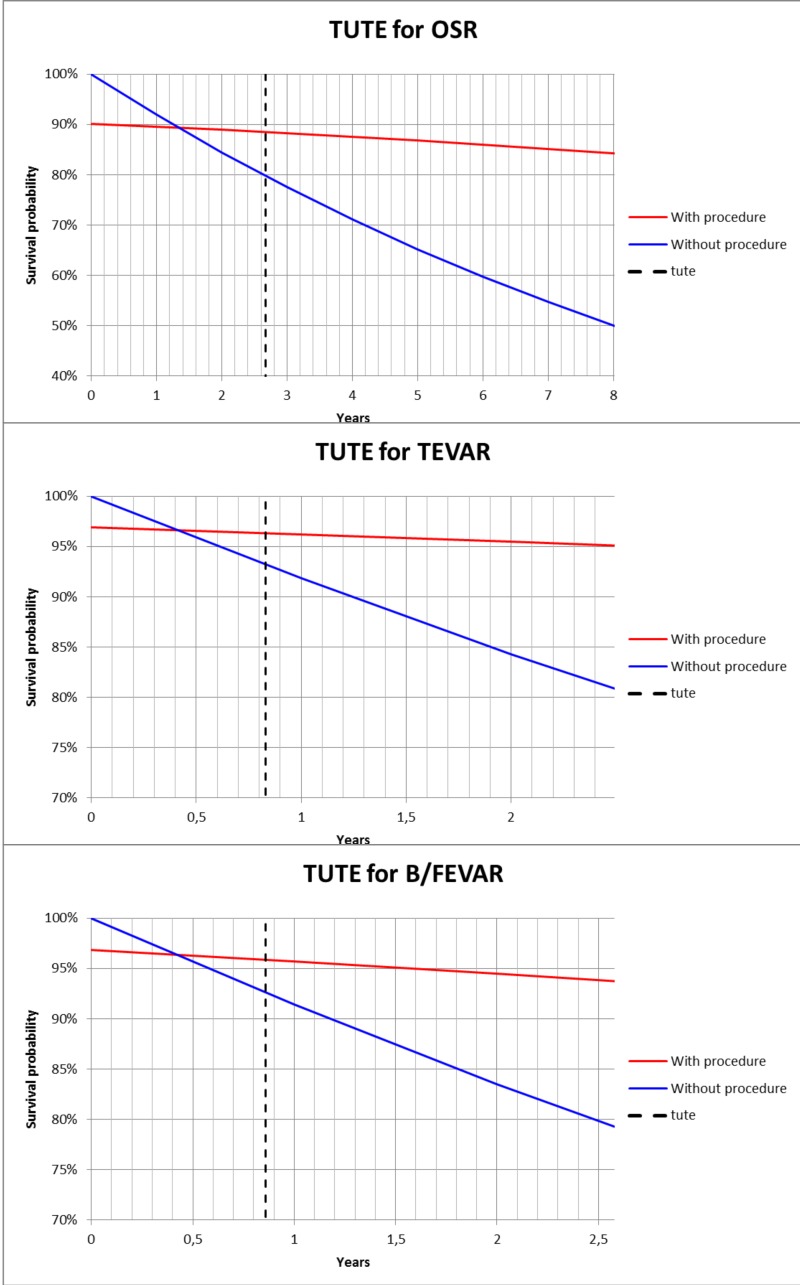
Time until Treatment Equipoise. **Results of TUTE analysis** for OSR (top), TEVAR (middle) and B/FEVAR (bottom) for CBAD.

The following parameters were entered in the TUTE analysis for each intervention: OSR (Male, 58 years, intervention mortality 9.9%, no intervention mortality 7.5%), TEVAR (male, 59 years, mortality intervention 3.1%, no intervention mortality 7.5%), and B/FEVAR (male, 65 years, mortality intervention 3.2%, no intervention mortality 7.5%). This resulted in TUTE for OSR of 2.7 years, for regular TEVAR this was 9.9 months and for B/FEVAR 10.3 months (Fig 2). This suggests TEVAR is the treatment that will have the earliest beneficial impact, compared to OSR and B/FEVAR. This is because TEVAR has lower operative risks compared to OSR. The available results of B/FEVAR are limited in current literature, making comparison vulnerable to bias. However, B/FEVAR seems to become more beneficial than just medical management after a similar timeframe as standard TEVAR, about 9–10 months after the incident dissection.

## Discussion

In this systematic review, short and long-term mortality rates of TEVAR for CBAD seemed to be favorable compared to those managed with OSR. To actually compare the results of B/FEVAR to the other two intervention strategies is challenging because of the small number of studies available. Most complications were observed after TEVAR, mainly related to the stent-graft, such as endoleak, rupture, and malperfusion. Although more reinterventions were required after TEVAR, the type of procedure was usually less invasive. A large percentage (>60.0%) of the reinterventions was represented by another endovascular procedure, an embolization or a ballooning of the stent-graft. Complications following OSR were usually more severe and the subsequent reintervention was frequently another open procedure. Complication rates after B/FEVAR were generally low, usually represented by endoleaks, and reintervention rates were high, always endovascular.

Our results show that it is difficult to distinguish which intervention is most suitable for CBAD. Currently, no randomized controlled clinical trials exist, mainly due to the rarity of the disease, to provide definitive evidence on optimal management strategy for CBAD. Therefore, comparison of observational data of these management modalities is important.

Actually, management of complicated acute and subacute type B dissection is usually performed by TEVAR while OSR is reserved for those patients affected by connective tissue disorders or with unsuitable anatomy for endovascular approach [1]. For CBAD patients this choice is more challenging, because other factors play a role in decision-making. In the chronic phase the TL is often small due to chronic compression of the lumen and scarring and thickening of the intimal flap occurs. Therefore, TL expansion and aortic remodeling is more challenging to accomplish when compared to treatment in the acute phase [1]. Moreover, the frequent involvement of the abdominal aorta in type B dissection explains why TEVAR for chronic dissections, although liberally used, is associated with high reintervention rates.

OSR is often used as the treatment of choice for CBAD, especially for extensive dissections involving visceral arteries and for patients that are deemed unsuitable for TEVAR. Besides several technical problems, as mentioned above, short landing zones or strong angulation in the arch could inhibit the use of endovascular techniques.

B/FEVAR allow for treatment of more challenging cases by endovascular means. This novel technique has been reported in highly selected cases in limited expert centers because of challenging issues related to narrow lumens, correct orientation of branches and fenestrations, and diminished sealing capacity in such setting. Because of limited reporting on B/FEVAR, it is difficult to compare it to standard TEVAR and OSR, and additional and long-term results are highly anticipated.

TUTE has been recently introduced to educate patients, but also to determine appropriate timing of an intervention[19]. Our analysis showed that the TUTE for regular TEVAR was 9.9 months, 10.3 months for B/FEVAR, and 2.7 years for OSR. Such results are in agreement with the increasing CBAD standard TEVAR management. The reason lies in the lower operative risk compared to OSR, associated with a relevant percentage of positive outcomes, despite higher rates of reintervention.

This systematic review has several limitations; first, we did not perform qualitative analyses. After careful consideration with our affiliated statistical center (Julius Support Center, UMC Utrecht, The Netherlands), it was considered to be not feasible and advisable to perform a meta-analysis. The heterogeneity among the data was too large, since all studies used different in- and exclusion criteria, diverse definitions, and reported different follow-up times. Furthermore, in many studies the original data were not present. Another limitation is that the rate of elective or urgent/emergent interventions differed fundamentally between studies, and a large number of studies did not report any procedural details. This might have caused differences in occurrence of complications and mortality.

### Conclusion

In conclusion, this systematic review suggests a limited early survival benefit of standard TEVAR over OSR for CBAD. The complication rates after TEVAR are higher, but the complications after OSR are usually more serious. Initial experiences with B/FEVAR show that this is a safe and feasible approach but long-term results are needed to compare it to OSR and standard TEVAR. Nevertheless, further development of dedicated branched and fenestrated stent-grafts for CBAD is needed to continuously improve their performance and broaden its indications. Until then, optimal treatment of CBAD remains debatable and merits a patient specific decision based on anatomy, life expectancy, general patient condition, and available expertise. TUTE seems a feasible and useful tool to better understand management outcomes of CBAD.

## Supporting Information

S1 Appendix Search strategy MEDLINE. (DOCX)

S2 Appendix PRISMA Checklist. (DOC)

S1 Table Demographics and OSR details. (DOCX)

S2 Table Demographics and TEVAR details. (DOCX)

S3 Table Demographics and B/FEVAR details. (DOCX)
