# Supplementary material for: Contemporary Management Strategies for Chronic Type B Aortic Dissections: A Systematic Review
Source: PLoS One. 2016 May 4;11(5):e0154930. doi: 10.1371/journal.pone.0154930 (PMC4856408; doi:10.1371/journal.pone.0154930)
Supplement: S1 Appendix — (DOCX) [file pone.0154930.s001.docx]

**Appendix 1.**

MEDLINE:

(Chronic[title] OR post-dissection[title]) AND (aortic[tiab] OR aorta[tiab]) AND (dissection[tiab] OR dissections[tiab) AND (type b[tiab] OR descending[tiab])
