# Supplementary material for: Contemporary Management Strategies for Chronic Type B Aortic Dissections: A Systematic Review
Source: PLoS One. 2016 May 4;11(5):e0154930. doi: 10.1371/journal.pone.0154930 (PMC4856408; doi:10.1371/journal.pone.0154930)
Supplement: S1 Table — (DOCX) [file pone.0154930.s003.docx]

**S1 Table. Demographics and OSR details**

|  | Andersen 2014[21] | Bashir 2014[22] | van Bogerijen 2015[29] | Conrad 2011[23] | Conway 2014[24] | Estrera 2015[25] | Fujikawa 2015 [30] | Goksel 2008[26] | Kouchoukos 2015[27] | Nozdrzykowski 2013[28] | Safi 2002[31] |
| --- | --- | --- | --- | --- | --- | --- | --- | --- | --- | --- | --- |
| OSR | 32 | 62 | 90 | 73 | 86 | 209 | 234 | 15 | 69 | 15 | 196 |
| CBAD definition | >2 weeks | NR | >2 weeks | NR | NR | >2 weeks | >2 weeks | >2 weeks | NR | NR | >2 weeks |
| FU in months (mea/med) | 34.0 (med) | 43.2 (mea) | 34.8 (med) | 53.0 (mea) | 55.2 (med) | 102.0 (med) | NR | 36.0 (mea) | 64.8 (mea) | 42.0 (med) | NR |
| Age in years (sd) | 55.0 (NR) | 52.4 (14.4) | 56.4 (12.8) | 64.5 (13.5) | 57.0 (NR) | 59.0 (NR) | 60.4 (13.0) | 62.0 (12.0) | 54.0 (16.0) | 61.0 (NR) | NR |
| Male | 27 (84.4) | 45 (72.6) | 70 (77.8) | 47 (64.4) | 59 (68.6) | 154 (73.7) | 187 (79.9) | 11 (73.3) | 53 (76.8) | 10 (66.7) | 153 (78.1) |
| HTN | 29 (90.6) | 37 (59.7) | 81 (90.0) | 67 (91.8) | 81 (94.2) | 187 (89.5) | 132 (56.4) | NR | 49 (71.0) | 15 (100.0) | 157 (80.1) |
| Diabetes | 1 (3.1) | 0 | 8 (8.9) | 5 (6.9) | NR | 23 (11.0) | 16 (6.8) | 2 (13.3) | 2 (2.9) | 0 | NR |
| Smoker | 20 (62.5) | 10 (16.1) | 56 (62.2) | 54 (74.0) | NR | NR | 126 (53.8) | NR | 29 (42.0) | NR | NR |
| Marfan | 9 (28.1) | NR | 12 (13.3) | 11 (15.1) | 7 (8.1) | 14 (6.7) | NR | 0 | 30 (43.5) | 2 (13.3) | NR |
| CKD | 4 (13.0) | 6 (9.7) | NR | NR | 11 (13.0) | 54 (26.0) | 16 (6.8) | 0 | 9 (13.0) | 4 (27.0) | NR |
| CAD | 8 (25.0) | NR | 11 (12.2) | 28 (38.4) | NR | 39 (18.7) | 45 (19.2) | NR | 10 (14.5) | NR | NR |
| Prior dissection | NR | NR | NR | NR | NR | NR | NR | NR | NR | NR | NR |
| Prior aneurysm | NR | NR | 4 (4.4) | 17 (23.3) | NR | 18 (8.6) | NR | NR | NR | NR | NR |
| Prior aortic surg | 21 (65.6) | NR | 28 (31.1) | NR | 29 (33.7) | 82 (39.2) | 138 (43.7) | NR | 54 (78.3) | 4 (26.7) | NR |
| Prior cardiac surg | NR | NR | 4 (4.4) | NR | NR | 50 (23.9) | NR | NR | NR | See prior aortic | NR |
| Elective | 26 (81.3) | 33 (53.2) | NR | 43 (58.9) | 77 (89.5) | 200 (95.7) | 216 (92.3) | NR | NR | NR | NR |
| Emergency | 1 (3.1) | NR | NR | NR | NR | 9 (4.3) | 18 (7.7) | NR | NR | NR | NR |
| Time from dissection to OSR | 31.0-43.0 (med) | NR | 32.4 (mea) | NR | NR | NR | 61.0 (mea) | NR | NR | NR | NR |
| CPB | NR | NR | 1 (11.1) | NR | NR | 206 (98.6) | NR | 13 (86.7) | 69 (100.0) | NR | NR |
| LHB | NR | NR | 19 (21.1) | NR | NR | NR | 223 (95.3) | NR | NR | NR | NR |
| (D)HCA | NR | NR | 70 (77.8) | NR | 36 (41.9) | 3 (1.4) | 6 (2.6) | NR | 69 (100.0) | 4 | NR |
| Spinal cord protection | 32 (100.0) | NR | 78 (86.7) | 51 (69.9) | 75 (87.2) | 209 (100.0) | NR | NR | 22 (31.9) | 13 | NR |

CAD= coronary artery disease; CBAD= chronic type B aortic dissection; CKD=chronic kidney disease; CPB=cardiopulmonary bypass; (D)HCA= (deep) hypothermic circulatory arrest; FU=follow-up; HTN=hypertension; LHB= left heart bypass; OSR= open surgical repair
