# Supplementary material for: Contemporary Management Strategies for Chronic Type B Aortic Dissections: A Systematic Review
Source: PLoS One. 2016 May 4;11(5):e0154930. doi: 10.1371/journal.pone.0154930 (PMC4856408; doi:10.1371/journal.pone.0154930)
Supplement: S2 Table — (DOCX) [file pone.0154930.s004.docx]

**S2 Table. Demographics and TEVAR details**

|  | Andacheh 2012[32] | Andersen 2014[21] | van Bogerijen 2015[29] | Chen 2013[33] | Czerny 2010[34] | Guangqi 2009[35] | Jia 2013[36] | Kang 2011[37] | Kato 2002[38] | Kim 2009[39] | Kitamura 2014[40] | Lee 2013[41] | Melissano 2008[42] | Nathan 2015[43] | Nozdrzykowski 2013[28] | Oberhuber 2011[44] | Parsa 2011[45] | Patterson 2013[52] | Sayer 2008[46] | Scali 2013[47] | Shimono 2002[48] | Song 2006[49] | Xu 2010[50] | Yang 2012[51] |
| --- | --- | --- | --- | --- | --- | --- | --- | --- | --- | --- | --- | --- | --- | --- | --- | --- | --- | --- | --- | --- | --- | --- | --- | --- |
| TEVAR | 73 | 44 | 32 | 56 | 14 | 49 | 208 | 76 | 14 | 72 | 53 | 71 | 11 | 47 | 32 | 19 | 51 | 195 | 40 | 80 | 13 | 17 | 84 | 28 |
| CBAD def | NR | >2 weeks | >2 weeks | >2 weeks | NR | >2 weeks | >2 weeks | >2 weeks | NR | >2 weeks | >2 weeks | >2 weeks | NR | >2 weeks | NR | >2 weeks | NR | > 2 weeks | >2 weeks | NR | NR | >2 weeks | >4 weeks | NR |
| FU in months (mea/med) | 18 (mea) | 34 (med) | 34.8 (mea) | 2.5 year (mea) | 34 (med) | 22.1 (mea) | 28.5 (mea) | 33.5 (mea) | 27 (mea) | 43 (med) | 7.5 year (mea) | 49.1 (med) | 12 (med) | 35.1 (mea) | 42 (med) | 13 (med) | 27 (mea) | 28.8 (mea) | NR | 26 (median) | 24.5 | 11 | 33.2 | 24.1 |
| Age in years (sd) | 58.0 (NR) | 59.0 (NR) | 69.2 (10.7) | 53.9 (10.7) | 63.0 (NR) | 57.1 (10.0) | 52.1 (21.8) | 61.5 (12.5) | 61.0 (14.0) | 55.0 (12.0) | 55.5 (13.1) | 55.1 (12.1) | 58 (NR) | 58.3 (11.7) | 62.0 (NR) | 60.0 (NR) | 57.0 (12.0) | 63,1 (NR) | 66.6 (11.9) | 60.0 (NR) | 60.3 (14.1) | 64.0 (14.0) | 53.3 (11.6) | 62.7 (12.6) |
| Male | 52 (71.2) | 28 (63.6) | 15 (46.9) | 44 (78.6) | 11 (78.6) | 46 (93.9) | 154 (74.0) | 49 (64.5) | 12 (85.7) | 47 (65.3) | 50 (94.3) | 50 (70.4) | 11 (100.0) | 35 (74.5) | 23 (71.9) | 17 (89.5) | 37 (72.5) | 161 (82.6) | 26 (65.0) | 70 (88.0) | 10 (76.9) | 10 (58.8) | 69 (82.1) | 24 (85.7) |
| HTN | NR | 43 (97.7) | 1 (3.1) | 49 (87.5) | NR | 47 (95.9) | 187 (89.9) | 75 (98.7) | NR | 67 (93.1) | NR | 58 (81.7) | 9 (81.8) | 41 (87.2) | 32 (100.0) | 18 (94.7) | 48 (94.1) | 182 (93.3) | 27 (67.5) | 76 (95.0) | 8 (61.5) | 16 (94.1) | 67 (79.8) | 26 (92.9) |
| Diabetes | NR | 5 (11.4) | 3 (9.4) | 6 (10.7) | NR | NR | NR | 8 (10.5) | NR | 10 (13.9) | NR | 4 (5.6) | 1 (9.1) | NR | 9 (28.1) | NR | 4 (7.8) | 22 (11.3) | NR | NR | 2 (15.4) | 2 (11.8) | 9 (10.7) | 4 (14.3) |
| Smoker | NR | 26 (59.1) | 22 (68.8) | 38 (67.9) | NR | NR | NR | 32 (42.1) | NR | 34 (47.2) | NR | 28 (39.4) | 6 (54.2) | NR | NR | 9 (47.4) | 27 (52.9) | 109 (55.9) | NR | 41 (51.3) | NR | 0 | 26 (31.0) | 12 (42.9) |
| Marfan | NR | 0 | 1 (3.1) | NR | NR | 1 (2.0) | NR | 2 (2.6) | NR | 2 (2.8) | 0 | 5 (7.0) | NR | NR | 0 | NR | NR | NR | 7 (17.5) | 7 (8.8) | NR | NR | 1 (1.2) | NR |
| CKD | NR | 7 (15.9) | NR | 3 (5.4) | NR | 3 (6.1) | 16 (7.7) | 14 (18.4) | NR | 4 (5.6) | NR | 4 (5.6) | 1 (9.1) | NR | 13 (40.6) | 6 (31.6) | 12 (23.5) | 21 (10.8) | NR | NR | 0 | 5 (29.4) | NR | 2 (7.1) |
| CAD | NR | 5 (11.4) | 8 (25.0) | 18 (32.1) | NR | NR | 25 (12.0) | 29 (38.2) | 1 (7.1) | 12 (16.7) | NR | 2 (2.8) | 3 (27.3) | NR | NR | 4 (21.1) | NR | 33 (16.9) | NR | 15 (18.8) | NR | 2 (11.8) | NR | 4 (14.3) |
| Prior dissection | NR | NR | NR | NR | 2 (14.3) | NR | NR | 17 (22.4) | NR | NR | NR | 15 (21.1) | NR | 13 (27.7) | NR | NR | NR | NR | NR | NR | NR | NR | NR | NR |
| Prior Aneurysm | NR | NR | NR | NR | NR | NR | NR | 12 (15.8) | 1 (7.1) | NR | NR | 2 (2.8) | NR | 8 (17.0) | NR | NR | NR | 22 (11.3) | NR | 8 (10.0) | NR | NR | NR | NR |
| Prior aortic surgery | NR | 12 (27.3) | 11 (34.4) | NR | 2 (14.3) | NR | NR | 29 (38.2) | 3 (21.4) | 3 (4.2) | 11 (20.8) | 15 (21.1) | 3 (27.3) | 29 (61.7) | 6 (18.8) | NR | 24 (47.1) | NR | 11 (27.5) | 26 (32.5) | 3 (23.1) | 2 (11.8) | NR | NR |
| Prior cardiac surgery | NR | NR | 1 (3.1) | NR | 1 (7.1) | NR | NR | NR | NR | 1 (1.4) | NR | 2 (2.8) | NR | NR | See prior aortic | NR | NR | NR | 3 (7.5) | NR | NR | NR | NR | NR |
| Elective | NR | 32 (72.7) | NR | NR | NR | NR | NR | NR | 14 (100.0) | NR | NR | NR | NR | NR | 20 (62.5) | NR | 38 (74.5) | 179 (91.8) | 35 (87.5) | NR | NR | NR | NR | NR |
| Emergency | NR | 1 (2.3) | NR | NR | NR | NR | NR | NR | NR | NR | NR | 2 (2.8) | NR | 2 (4.3) | 6 (18.8) | NR | 1 (2.0) | 15 (7.7) | 5 (12.5) | NR | NR | 2 (11.8) | NR | 1 (3.6) |
| Time from dissection to TEVAR | NR | 17 (median) | 20,3 (mean) | 0,8 (mean) | 19 (median) | NR | 0,8 (median) | 25 (mean) | 35 (mean) | NR | 16,8 (mean) | 16 (median) | NR | 53,8 (mean) | NR | 36 (median) | 46,2 (mean) | NR | 16 (median) | NR | NR | 13,9 (mean | 49,8 (mean) | NR |
|  |  |  |  |  |  |  |  |  |  |  |  |  |  |  |  |  |  |  |  |  |  |  |  |  |
| Type stent | Medtronic Talent, Valiant Captivia | NR | Gore TAG, Cook Zenith TX2, Medtronic Talent | Medtronic Talent, Willis Microport | Medtronic Valiant/ Talent, Bolton Relay | Medtronic Talent, Cook Zenith, Lifetech Ankura, Miroport Aegis | Medtronic Valiant, Cook Zenith TX2, Microport Hercules | Gore TAG, Cook Zenith, Homemade, Medtronic Talent, Multiple | NR | Biotech | Handmade, MNRK | Biotech SEAL, Taewoong, Medtronic Valliant, Cook Zenith TX2 | Cook Zenith, Gianturco | Cook Zenith TX2, Gore TAG, Medtronic Talent | NR | Gore TAG, Gore C TAG, Medtronic Captivia/Valiant, Cook Zenith | Gore TAG, Cook Zenith, Medtronic Talent | Medtronic Talent, Medtronic Valliant | Medtronic Valiant/Talent, Gore Excluder | Gore TAG, Cook Zentih TX2 | Cook Gianturco | AneurRX, Medtronic Talent | MedtronicTalent, Endofit, Microport Hercules, Vasoflow Vascore, Grikin Grikin | Cook Zenith |

CAD= coronary artery disease; CBAD= chronic type B aortic dissection; CKD=chronic kidney disease; FU=follow-up; HTN=hypertension; TEVAR=thoracic endovascular aortic repair
