# Supplementary material for: Contemporary Management Strategies for Chronic Type B Aortic Dissections: A Systematic Review
Source: PLoS One. 2016 May 4;11(5):e0154930. doi: 10.1371/journal.pone.0154930 (PMC4856408; doi:10.1371/journal.pone.0154930)
Supplement: S3 Table — (DOCX) [file pone.0154930.s005.docx]

**S3 Table. Demographics and B/FEVAR details**

|  | Kitagawa 2013^a^ [53] | Kitagawa 2013^b^ [53] | Oikonomou 2014 [54] |
| --- | --- | --- | --- |
| B/FEVAR | 15 | 15 | 31 |
| CBAD def | NR | NR | NR |
| FU in months (mea/med) | 20.4 (mea) | 20.4 (mea) | 17.0 (mea) |
| Age in years (sd) | 58.0 (11.0) | 74.0 (8.0) | 65.0 () |
| Male | 14 (93.3) | 12 (80.0) | 25 (80.6) |
| HTN | 13 (86.7) | 12 (80.0) | 27 (87.1) |
| Diabetes | 1 (6.7) | 3 (20.0) | 4 (12.9) |
| Smoker | 10 (66.7) | 10 (66.7) | 22 (71.0) |
| Marfan | 4 (26.7) | 0 | 1 (3.2) |
| CKD | 2 (13.3) | 3 (20.0) | 10 (32.3) |
| CAD | 2 (13.3) | 11 (73.3) | 20 (64.5) |
| Prior dissection | NR | NR | NR |
| Prior Aneurysm | NR | NR | NR |
| Prior aortic surgery | NR | NR | NR |
| Prior cardiac surgery | NR | NR | NR |
| Elective | 15 (100.0) | 15 (100.0) | 31 (100.0) |
| Emergency | 0 | 0 | 0 |
| Time from dissection to B/FEVAR in months | NR | NR | 31.0 |
| Technical success in % | 100.0 | 100.0 | 93.5 |
| Type stent | Cook | Cook | Cook |

B/FEVAR=branched and fenestrated thoracic endovascular aortic repair; CAD= coronary artery disease; CBAD= chronic type B aortic dissection; CKD=chronic kidney disease; FU=follow-up; HTN=hypertension

^a^Extensive dissection cohort (Type II/III) ^b^Focal dissection cohort (without visceral involvement)
